# Automation of Detector Array Design for Baggage X-Ray Scanners

**DOI:** 10.3390/s25247550

**Published:** 2025-12-12

**Authors:** Krzysztof Dmitruk

**Affiliations:** Institute of Computer Science and Mathematics, Maria Curie-Sklodowska University, Akademicka 9, 20-031 Lublin, Poland; krzysztof.dmitruk@umcs.pl

**Keywords:** X-ray scanner, baggage scanner, detector array alignment, X-ray geometry modeling, X-ray scanner design

## Abstract

Geometric inaccuracies in the design of X-ray baggage scanners can lead to significant image artifacts, such as banding and discontinuities, which compromise security screening effectiveness. Although comprehensive commercial solutions are available, constructing a custom X-ray scanner requires the precise alignment of detector arrays. This is a complex and time-consuming process when performed manually. The core of the proposed method is a computational model that calculates the optimal position and orientation for each detector card based on user-defined scanner dimensions and hardware parameters. To validate the geometry created with this method, its performance was compared against flat and arc-shaped geometries. The results demonstrate that the proposed method successfully generates geometries that produce continuous and artifact-free images. The study concludes that the developed software tool provides a robust and practical solution, significantly simplifying the complex task of scanner construction and accelerating the development of reliable, custom X-ray inspection systems.

## 1. Introduction

X-ray scanners used for baggage inspection are tools for ensuring security in airports, courthouses, and other public spaces. They are capable of revealing the contents of luggage without opening it and are used to detect dangerous items and contraband. The result of such a scanner’s operation is most often a 2D image which is then interpreted by an operator.

**Figure 1:**
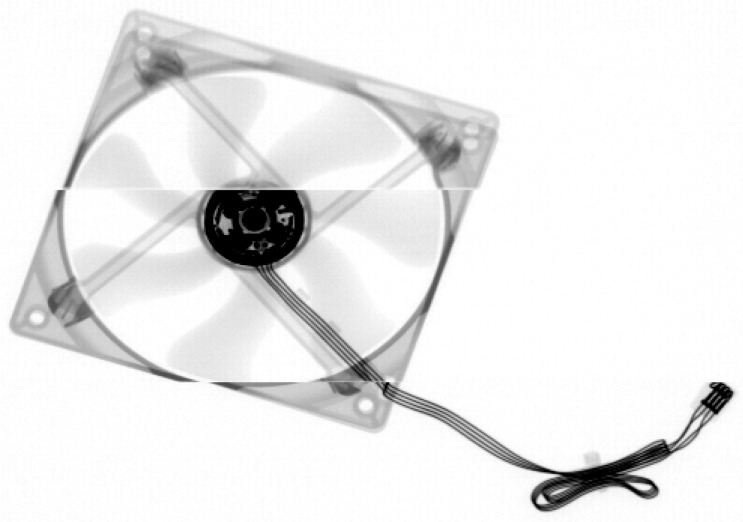
Image acquired from a X-ray scanner with improper geometry (intentionally recreated using the laboratory scanner described in Section 6.2).

The motivation for this work came from author’s participation in the project to create a baggage X-ray scanner integrated into a vehicle. The scanner was designed and built by a company specializing in vehicle conversions, which had little experience in constructing X-ray scanners. The device produced low-quality images, with noticeable banding and visible artifacts along the scan axis as seen in Figure 1. It was found that the scanner geometry had been poorly designed. To compensate for these defects, the detectors were manually realigned. The resulting image also required cropping and translation of its sections, while missing lines were reconstructed using the inpainting method [1]. Although the final image quality was accepted by the client, the author considers it to have remained suboptimal. This experience made it clear that designing a geometrically proper scanner is a complex task that requires a lot of precision in both design and construction.

The main goal of this study was to create a parameterizable method for construction of the detector card array—a part of the scanner used for the X-ray image acquisition. For this task, a software tool was developed. It computes a list of detector card positions based on the scanner dimensions and the hardware parts parameters specified by the user. This model can then be used to design a geometrically accurate detector array.

**Figure 2:**
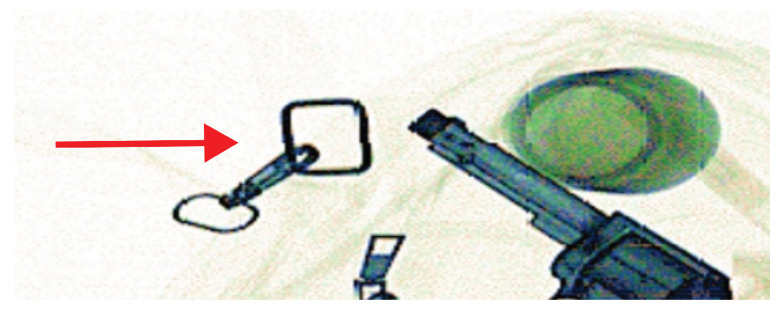
A fragment of an image from the PIDray database [4], containing a discontinuity along the horizontal line. The arrow indicates the location of the discontinuity.

The challenge of constructing a geometrically accurate X-ray baggage scanner is not new. Functioning solutions clearly exist, as evidenced by the performance of commercial scanners. Image databases [2,3,4] contain thousands of visually correct images, although images with discontinuities can also be found, as shown in Figure 2.

In most papers concerning X-ray scanners, it is rightly assumed that the images produced are geometrically accurate. However, the issue of the scanner’s mechanical and geometric correctness, while niche, is crucial from the standpoint of image quality. Material classification algorithms, both classical [5] and AI-based [6,7], take into account the neighborhood of the classified region and may therefore be misled by abrupt pixel value changes unrelated to the scanned object. The most widely addressed related topic in the literature is the correction of instrument misalignments in computed tomography (CT). It is well known that scintillator panels are subject to slight geometric distortions. Weiss [8], Luethi [9], and Hardner [10] present possible correction algorithms. CT-related methods, however, require multiple images of the same region and therefore cannot be applied in scanners that produce only a single image. For the correction of single-image X-ray scans, Fotouhi [11] proposed a convolutional network based method for stitching mismatched regions across consecutive detectors.

The manufacturers of commercial scanners do not disclose their solutions, leaving it unclear whether detector arrays are designed manually, generated automatically, or developed using a hybrid of these approaches. To the best of the author’s knowledge, the concept of programmatically creating detector array geometries has not been previously described in this context. In particular, the presented software, which can be parameterized to support scanners of user-defined size and hardware configuration, is novel.

In the design of custom scanners e.g., research applications, the use of an automated detector array configuration method is particularly advantageous. Manual adjustment of detector positions is both time-consuming and impractical due to the hazardous nature of X-ray radiation. The inability to reposition detectors and simultaneously observe the resulting changes in the acquired image further limits the effectiveness of manual methods.

A further benefit of constructing a detector array is the complete knowledge of the device geometry, which can be valuable for object reconstruction. When a device lacks explicit geometry information, complex methods must be employed to estimate it. Senchukova [12] presents an approach to addressing this problem in an industrial context.

This paper begins with a discussion of how image formation in an X-ray scanner is influenced by its geometric configuration. This is followed by a detailed explanation of the proposed method for generating detector arrays, and concludes with results obtained from both simulation and testing using physical detectors.

## 2. Image

In an X-ray scanner, the image is formed through the absorption of X-ray radiation by the scanned object [13]. The radiation is emitted from an X-ray tube and is registered by one or more detectors.

Depending on the scanner’s application, different types of imaging projections are used. In analog scanners, the imaging plane was a flexible film that could be shaped as needed, while the X-ray tube emitted radiation in the form of a conical beam. Modern digital detectors are typically flat panels. In systems where the setup is stationary, the entire image is captured at once on a rectangular detector. Such scanners are commonly used in medical imaging [14].

In baggage scanners, as well as in scanners used for non-destructive testing in quality assurance applications, a moving setup is typically employed. The object moves on a conveyor belt in the area between the X-ray tube and the detector. This setup enables a throughput of up to 1500 bags per hour [15], which is required for this application. Unlike the previously described stationary system, the detector here is linear in shape, and the photon beam emitted by the tube forms a fan-shaped pattern. The detector records the energy carried by the photons over a time interval known as the integration time. This data forms a single row in the resulting two-dimensional image. The movement of the conveyor belt causes successive regions of the scanned object to pass between the X-ray source and the detector, until the entire object has been fully scanned [16].

Due to the described motion, the resulting image exhibits a hybrid geometry: it is an orthographic projection along the conveyor belt direction, and a perspective projection along the axis perpendicular to it [17]. For the scanner operator, this is an advantage, as rotating the object may reveal components that were previously overlapping [18]. The application of a collimated fan-beam is also important in baggage scanners, as it allows for more effective suppression of radiation within the scanner enclosure [19]. This enables the scanners to operate safely in environments where people are present.

Detector modules used in X-ray baggage scanners are manufactured in a range of lengths. In the experiments conducted for this paper, compact 100 mm-long modules were employed. Used modules are insufficient to capture the full projection of a typical baggage. Longer modules extending to several hundred millimeters are available, but their use is limited by higher cost and reduced flexibility in geometry design. Therefore, scanners combine multiple modules into detector arrays or matrices, with each subsequent module covering the area not visible to its neighbor [20]. This modular approach allows scaling of scanners from compact systems for postal envelopes to large installations capable of inspecting air cargo or even shipping containers.

## 3. Geometric Model

The arrangement of detectors directly impacts the appearance of the resulting image. This section presents and discusses three potential configurations of the detector array.

The first and most straightforward approach is defined by placing all detectors along a single line, typically oriented perpendicularly to the direction of object movement. This geometry is referred to as flat in this paper. Several advantages are offered by this configuration: mechanical simplicity, no need for precise angular alignment, and independence from the source position. As long as the X-ray beam is directed toward the detector array, a coherent image can be produced, regardless of the angle or position of the source. A key drawback, however, is the progressively decreasing projection arc length on detectors located farther from the center. In the reconstructed image, this results in visual distortion [21], which must be corrected algorithmically. Furthermore, objects exposed to X-rays at large incidence angles are blurred as a result of parallax error [22].

In the second type of geometry, the detectors are arranged along an arc. In this configuration, a corresponding point on each detector (e.g., the center) is positioned at an equal distance from the X-ray source. In this way, the issues associated with the linear configuration are resolved. This geometry is referred to as arc-shaped in this paper. Because of its geometrical optimality, this arrangement is widely employed in CT scanners. In CT systems, the detector array and the X-ray source are rotated together around the center point between them [23]. Through this rotation, data can be captured over a full 360° angular range, which is not possible in a static setup.

In baggage scanners, by contrast, the detector array and the X-ray source are typically stationary, and the scanning area can only be extended by increasing the number of detectors. Since baggage scanners are designed primarily for objects of rectangular shape, equal distances between the source and all detectors could only be maintained if the overall size of the scanner were increased. In such systems, distortion correction must be applied, as the outer edges of each flat detector module are positioned slightly farther from the source than their centers. In this configuration, the position of the X-ray source is strictly defined, and any deviation from the designed location results in partial obstruction of neighboring detectors along the beam path.

The third type of geometry is a modification of the arc-based arrangement. Its primary design principle is that each detector is oriented such that the X-ray beam strikes its center at a perpendicular angle. In contrast to the previous configuration, a constant distance between the detectors and the X-ray source is not maintained. Instead, the detectors are placed so that the edges of their active areas closest to the source lie on a common straight line. This ensures more energy for each detector than in the arc-based geometry. Also, the array can be integrated into a compact housing. Certain drawbacks, however, are introduced. Abrupt changes in signal levels between neighboring detectors are caused by different degrees of X-ray attenuation by air over varying path lengths. This effect can be effectively addressed through calibration procedures such as flat-field correction [24]. This geometry is commonly observed in commercial scanner systems, is depicted in multiple expired and active baggage scanner-related patents [25,26], and is employed in complex applications such as X-ray stereography [27]. In subsequent sections, this configuration is called the compact geometry.

Detector arrays based on all three described geometries were constructed and employed with the same scanner. The comparative results are presented in Section 6.

## 4. Model Setup

### 4.1. Input Data

The model construction requires data specifying the position of the X-ray source, along with the location and dimensions of the detector array. The following coordinate system is adopted:

- *x*—scanner width.
- *y*—scanner length, which corresponds to the direction of conveyor belt movement.
- *z*—scanner height.

All elements of the model lie on a common plane with a constant *y*. Therefore, this coordinate will not be used in the process of modeling the detector array geometry. It will be utilized in the interpretation of the results in Section 6.

The initial step involves defining the position of the X-ray tube, which determines the location of the focal spot. This is the area on the tube’s anode where the electron beam from the cathode is concentrated, resulting in the generation of X-rays [28]. The size of focal spot is negligible for the geometry calculation (e.g., 33 μm in the tube used in this paper). For modeling purposes, the focal spot is approximated as a point source.

Another characteristic of the X-ray source is the angular range over which radiation is emitted. For systems employing linear detectors, fan-beam X-ray tubes are standard, as they generate radiation along a single axis. This angular range defines the region within which the detectors can receive any radiation from the source. Manufacturers typically guarantee consistent performance characteristics across the entire fan beam [29]. Detailed information regarding the focal spot position and emission angles is available in the technical documentation provided by the manufacturer.

On the opposite side of the scanner’s tunnel, the designer should position an array of X-ray detectors. The array’s location, overall size, and the dimensions of its individual detector card determine the total number of detectors that can be integrated.

### 4.2. Result Format

The output data is a vector of detector positions. Each detector is defined by two 2D points that delimit the segment representing the scintillation layer of the detector. Based on this data, a physical model of the detector array can be created, ready for the installation of individual detectors.

### 4.3. Geometry Visualization

**Figure 3:**
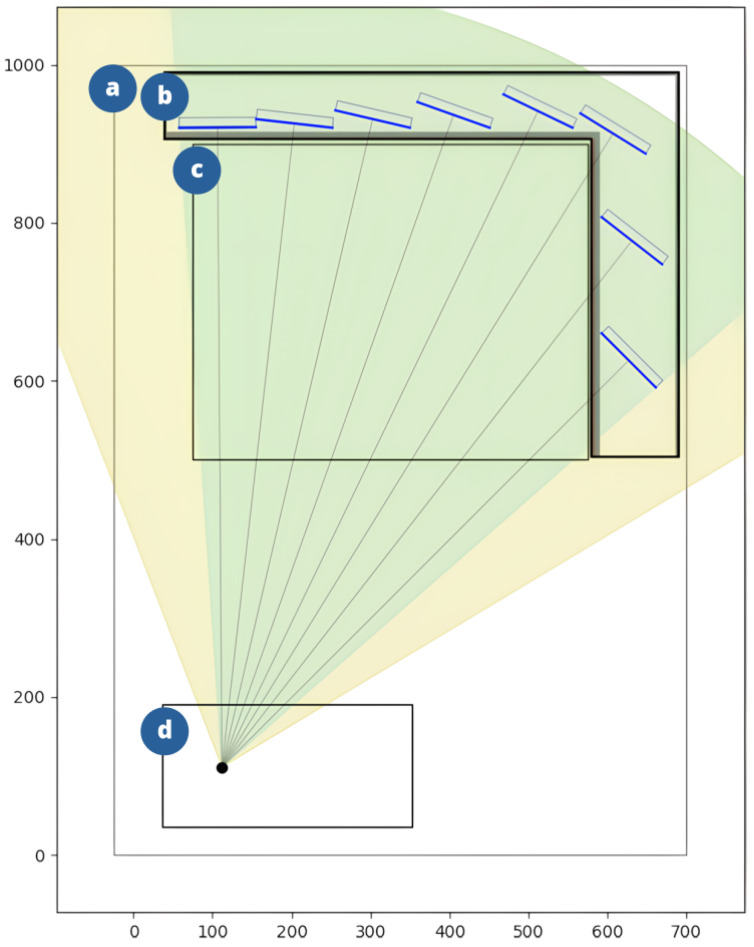
Diagram of a geometry model with dimensions, in millimeters, corresponding to a typical baggage scanner with a L-shaped detector array. The visualization comes from an application that is part of this paper. The following elements are present: (**a**) Scanner housing; (**b**) Detector array, with detectors located inside; the blue segment indicates the position of the active area of each detector, and the gray segment indicates the position of the card to be attached to the array; (**c**) Scanner tunnel; (**d**) X-ray tube, with the black dot inside representing the focal spot. The yellow circular sector represents the area of radiation, and its green part is the area where it overlaps with the region registered by the detectors.

Visualizing both the input configuration and the resulting geometry is useful for developing and validating the scanner model. The software created as part of this paper provides a graphical representation of a scanner’s detection plane. The geometry visualization as seen in the application is presented in Figure 3.

While the dimensions of the scanner housing and the tunnel are not directly used in calculations, they offer visual context that is helpful for understanding the physical constraints of the system and validate if the expected region is covered by the detector array.

The X-ray source is described by its physical dimensions, within which the focal spot and the angular emission range are fixed. The source can be translated and rotated allowing dynamic updates to the beam direction.

The position and size of the detector array are fully configurable. The detectors within the array are depicted as segments representing their scintillating layers As the relative positions of the source and detector array change, the detector layout is updated in real time. The first detector is positioned at the left edge of the array, and an offset parameter allows fine-tuning of the detector placement.

A central element of the visualization is the rendering of the angular detection range, shown as a sector originating from the focal spot. This makes it easy to verify whether the tunnel is fully covered by the beam and whether all detectors are exposed to the X-ray beam.

## 5. Method

In this section, the method for constructing the compact geometry is presented (with the exception of Section 5.3, where the arc-based geometry is discussed). The description of the algorithm is divided into stages. Initially, the objective is to construct a linear detector array. After discussing this case, the algorithm will be extended to support L-shaped and U-shaped configurations.

In the following steps, the linear array is treated as a segment between two points, $A_{L}$ and $A_{R}$, where $A_{L}$ is the lower-left corner and $A_{R}$ is the lower-right corner of the detector array. The region intended to be covered by detectors is defined by an arc extending between points $A_{L}^{\prime }$ and $A_{R}$, with the arc centered at point *F*, where $A_{L}^{\prime }$ is the point $A_{L}$ translated by a offset value along the *x* axis and *F* is the focal spot of the X-ray tube.

The angle formed between point *F* and point $A_{L}^{\prime }$ defines the starting angle of the arc to be covered by the detectors and is denoted as $\theta_{0}$. To determine the position of each detector, it is necessary to compute the angle that marks the beginning of its detection range within the arc. For the first detector, this starting angle is $\theta_{0}$.

Once the position of the *i*-th detector has been calculated, the algorithm returns the angle $\theta_{i+1}$, which represents the end of the current detector’s coverage also being the starting angle for the next detector. This process continues until the next hypothetical detector n+1 would extend, even partially, to the right of point $A_{R}$.

In this way, the total number of detectors *n* is determined, as well as the final coverage angle $\theta_{n}$, which marks the end of the detectable arc region.

### 5.1. Determining the Position of a Detector

The final representation of each detector’s position is defined by two points, $P_{L}$ and $P_{R}$, which correspond to the left and right endpoints of the segment representing the scintillation layer of the detector. In the case of a linear detector array, the position of each individual detector is computed based on the following parameters:

- *ℓ*—the physical width of the detector, determined by the hardware specification.
- $z_{0}$—the *z*-coordinate shared by points $A_{L}$, $A_{L}^{\prime }$, and $A_{R}$, representing the vertical position of the lower edge of the detector array.
- $\theta_{i}$—the angle that specifies the start of the arc segment to be covered by the *i*-th detector.

The endpoints of a detector can also be denoted based on their distance from the lower edge of the array: $P_{N}$, the point closer to the reference line $z_{0}$, and $P_{F}$, the point farther from it.

It should be determined which of the two possible configurations applies:

(1) $$\left(P_{L},P_{R}\right)=\left(P_{F},P_{N}\right),$$

or

(2) $$\left(P_{L},P_{R}\right)=\left(P_{N},P_{F}\right).$$

There is also a special case in which the detector is parallel to the line $z_{0}$. The position of such a potential detector is determined as follows. Point $P_{L}$ is defined as the intersection of the line $z_{0}$ and the ray originating from point *F* at an angle $\theta_{i}$. Then, point $P_{R}$ is computed as the translation of $P_{L}$ by the vector $\vec{\left(\ell ,0\right)}$. The center of the detector, $P_{\mathrm{avg}}$, is the midpoint between $P_{L}$ and $P_{R}$.

If $P_{\mathrm{avg}}.x\;=\;F.x$, then the points $P_{L}$ and $P_{R}$ define the final detector position. If $P_{\mathrm{avg}}.x\;<\;F.x$, this implies that Equation (1) holds, and the algorithm proceeds as described in Section 5.1.1. If $P_{\mathrm{avg}}.x\;>\;F.x$, then Equation (2) holds, and the next steps follow the procedure outlined in Section 5.1.2. Both variants are based on the bisection method.

#### 5.1.1. Translation-Based Fitting

This variant determines the position of point $P_{F}$ by translating it along the ray originating from point *F* and extending at an angle $\theta_{i}$. The first step is to compute the range of potential positions for $P_{F}$. This requires identifying two points lying on the ray:

- $P_{\min}$—the point where the ray intersects the line $z_{0}$.
- $P_{\max}$—the point located at a distance *ℓ* from $P_{\min}$ along the direction of the ray.

Next, the point $P_{F}$ is calculated as the midpoint between $P_{\min}$ and $P_{\max}$. The point $P_{N}$ lie at a distance *ℓ* from $P_{F}$ and is also located on the line $z_{0}$. These constraints are enough to determine the position of point $P_{N}$.

The points $P_{N}$ and $P_{F}$ define a potential position of the detector. It is necessary to verify the angle at which the detector is oriented relative to point *F*. To this end, the slopes of the following lines are computed:

- $a_{\mathrm{card}}$—for the line passing through the points $P_{N}$ and $P_{F}$.
- $a_{\mathrm{tube}}$—for the line connecting point *F* with the midpoint $P_{\mathrm{avg}}$ between $P_{N}$ and $P_{F}$.

Based on these slopes, the alignment indicator *d* is computed:

(3) $$d=a_{\mathrm{card}}\;a_{\mathrm{tube}}+1.$$

Indicator *d* is a signed scalar measure of how far and in which direction the detector card deviates from being perpendicular to the ray direction from the focal spot. Two lines are perpendicular when d=0. The sign of *d* determines which side of the optimal position the current detector lies on.

**Figure 4:**
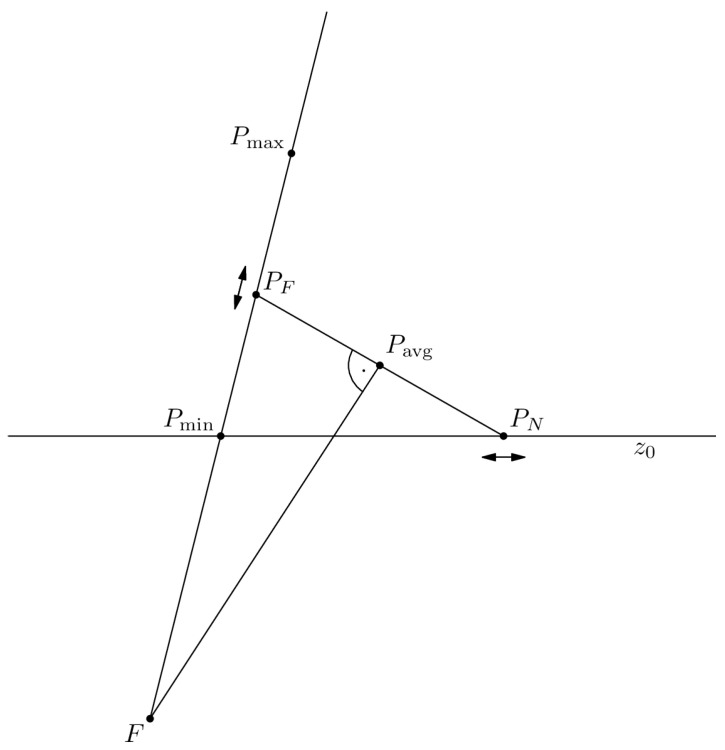
Scheme of the translation-based fitting. Points $P_{\min}$ and $P_{\max}$ are at their initial placement. Points $P_{N}$ and $P_{F}$ are at their final placement because segments $\bar{P_{N}P_{F}}$ and $\bar{FP_{\mathrm{avg}}}$ are perpendicular to each other. The arrows show how the points $P_{F}$ and $P_{N}$ may have been shifted while maintaining a constant distance *ℓ* between each other.

If d<0, then in the next step $P_{F}$ replaces $P_{\min}$. Otherwise, $P_{F}$ replaces $P_{\max}$. Subsequent iterations proceed analogously. The loop terminates when the change in *d* between two successive steps falls below the fixed threshold ε. Figure 4 presents the position of points important in the translation-based fitting process for a single detector card.

#### 5.1.2. Rotation-Based Fitting

This variant assumes that the point $P_{N}$ is fixed, and the goal is to adjust the position of the point $P_{F}$ accordingly.

The first step is to determine the position of $P_{N}$, which lies at the intersection of a ray originating from point *F* at angle $\theta_{i}$ and the line $z_{0}$. The point $P_{F}$ is located at a distance *ℓ* from $P_{N}$, at an angle within the range from $\alpha_{\min}=0^{\circ }$ (parallel to the line $z_{0}$) to $\alpha_{\max}=90^{\circ }$. The exact angle is computed using the bisection method.

The angle $\alpha_{\mathrm{avg}}$ is calculated as the arithmetic mean of $\alpha_{\min}$ and $\alpha_{\max}$. From point $P_{N}$, a segment of length *ℓ* is drawn at angle $\alpha_{\mathrm{avg}}$, and its endpoint defines point $P_{F}$.

**Figure 5:**
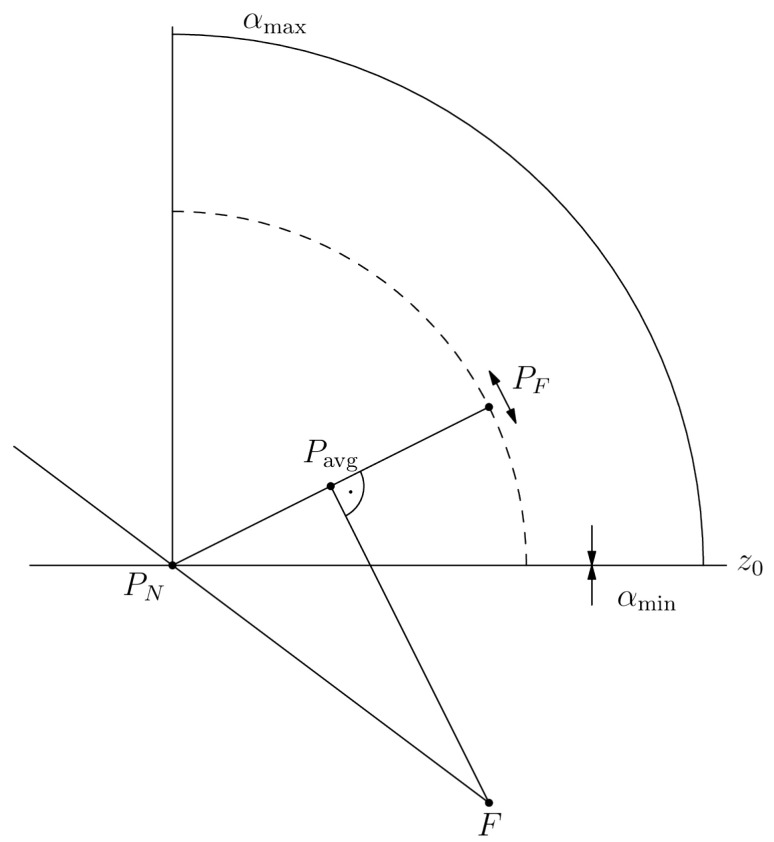
Scheme of the rotation-based fitting. The arrows show how the points $P_{F}$ may have been rotated while maintaining a constant distance *ℓ* from the stationary point $P_{N}$.

As in the previous variant, the midpoint $P_{\mathrm{avg}}$ is used to compute the alignment indicator *d* according to Equation (3). If d>0, the value of $\alpha_{\mathrm{avg}}$ replaces $\alpha_{\min}$ in the next iteration. Otherwise, it replaces $\alpha_{\max}$. As in the previous case, the loop continues until the difference in *d* values between two consecutive steps is smaller than a given threshold ε. Figure 5 presents the final step in the rotation-based fitting process for a single detector card.

### 5.2. L- and U-Shaped Array

A linear detector array alone does not solve the problem of maintaining compact scanner dimensions. To address this limitation, the array can be extended with one or two downward-facing arms, resulting in an inverted L-shaped or U-shaped configuration, respectively.

#### 5.2.1. Right Arm

If the array is to be extended with a right arm, its dimensions are defined by introducing the point $R_{B}$, which is the left (i.e., closer to the X-ray source) bottom point of the array’s right arm. This point replaces $A_{R}$ as the boundary for placing detectors, while $A_{R}$ becomes the array’s bending point.

If the angle $\theta_{i}$ is smaller than the angle defined by point $A_{R}$, the *i*-th detector will be located within the arm. In this case, the condition $\left(P_{L},P_{R}\right)=\left(P_{N},P_{F}\right)$ is always satisfied. Otherwise, the detectors would be oriented outward from the scanner.

The point $P_{N}$ lies on the vertical line $x_{R0}$, which is shared by the points $A_{R}$ and $R_{B}$. The method described in Section 5.1.2 is used to compute the position of $P_{F}$, with slight modification: due to the rotation of the entire detector assembly, the initial angle values are set to $\alpha_{\min}=270^{\circ }$ (parallel to line $x_{R0}$) and $\alpha_{\max}=0^{\circ }$.

#### 5.2.2. Left Arm

The array can also be be extended with a left arm. To maintain compatibility between the configurations with and without the left arm, the point $A_{L}^{\prime }$ remains the starting position. Consequently, a loop traversing angles in the counterclockwise direction is used to populate the left arm.

In the left arm, the condition $\left(P_{L},P_{R}\right)=\left(P_{F},P_{N}\right)$ is always satisfied. However, because of the counterclockwise iteration, the method described in Section 5.1.2 can also be applied here. As in the previous case, the point $L_{B}$, which is the bottom-right point of the left arm, is used as a detector array boundary.

If the value of $\theta_{i}$ is greater than the angle defined by point $A_{L}$, the *i*-th detector will be placed within the arm. The point $P_{N}$ will then lie on the line $x_{L0}$, which is shared by points $A_{L}$ and $L_{B}$.

In this case as well, the rotation of the detector system must be considered. Therefore, the initial range for the bisection method is: $\alpha_{\min}=270^{\circ }$ (parallel to line $x_{L0}$) and $\alpha_{\max}=180^{\circ }$.

The presence of both left and right arms within a single array does not impose any additional constraints.

#### 5.2.3. Right Corner Detector

Using the previously described method, a correct detector array can be constructed. The position of a corner detector, normally placed along the horizontal axis $z_{0}$, can be optimized by shifting it closer to point $A_{R}$ while remaining within the boundaries of the array.

The first step is to determine whether the *i*-th detector is a corner detector. This occurs when the angle θ corresponding to point $A_{R}$ lies between angles $\theta_{i}$ and $\theta_{i+1}$. In such a case, the detector calculated as in Section 5.1.1 is not added to the array. Instead, the goal is to find the position of a detector whose starting angle is $\theta_{i}$ and which is tangent to point $A_{R}$. This is achieved using an algorithm based on the method described in Section 5.1.1. According to that procedure, the placement range of point $P_{F}$ must first be defined. The point $P_{\max}$ is computed as before, since even a minimal overstep beyond the array’s corner (bend point) must be considered. The definition of point $P_{\min}$ is different though. It is now determined along the ray extending from *F* to $A_{R}$, placed on the line $x_{R0}$. If this boundary is crossed, the detector would lie entirely within the right arm, as described in Section 5.2.1.

The method of computing the potential point $P_{N}$ is also modified. For each midpoint $P_{F}$ between $P_{\min}$ and $P_{\max}$, a line is drawn connecting $P_{F}$ and $A_{R}$. Along this line, a segment of length *L* is marked starting from $P_{\mathrm{avg}}$ and containing point $A_{R}$. The second endpoint of this segment defines point $P_{N}$.

The remaining steps proceed as described in Section 5.1.1, including verification of perpendicularity and possible repetition of the bisection step.

#### 5.2.4. Left Corner Detector

If the left arm is enabled, the left corner detector must also be considered. Because of the counterclockwise order of detector placement described in Section 5.2.2, the translation-based method is also applied to add this detector. The procedure described in this section is a mirror reflection of the approach used for the right corner detector in Section 5.2.3.

Since the initial angle $\theta_{0}$ corresponds to the first detector on the left in the horizontal section of the array, the only possible left corner detector is the one with index i=−1. This situation arises when the angle formed by a ray extending from point *F* to $x_{L0}$ lies between $\theta_{-1}$ and $\theta_{0}$. In this case, the −1-th detector is treated as a corner detector. The point $P_{\max}$ remains unchanged, while $P_{\min}$ is determined along the ray extending from *F* to $A_{L}$ and placed on the line $x_{L0}$. All remaining steps are carried out analogously to the procedure described in Section 5.2.3.

This concludes the description of constructing the compact geometry.

### 5.3. Arc Mode

Arc mode is an independent algorithm designed to implement the second version of the geometry described in Section 3. This algorithm operates exclusively with a linear detector array (without arms), since this configuration is not expected to have practical applications in large scanners.

The first step in generating the geometry is to define the arc along which the detector edges will be placed. Between the points $A_{L}$ and $A_{R}$, the one located farther from point *F* is selected. This guarantees that all detectors are positioned above the line $z_{0}$. The resulting distance defines the radius *r* of the arc. The initial angle $\theta_{0}$ of the detection range is determined at the intersection of the vertical line passing through A’L.x with the circle centered at *F* and radius *r* within the array region. This intersection defines point $P_{L}$. Next, at a distance of *ℓ* clockwise from $P_{L}$ along the same circle, point $P_{R}$ is located. The points $P_{L}$ and $P_{R}$ define the target edges of a single detector. Each subsequent *i*-th detector starts at angle $\theta_{i-1}$. If this angle exceeds that defined by the right edge of the detector array, the detector is not added and the algorithm terminates.

Although the geometry described in this section serves a supplementary role and was primarily used for debugging purposes, it was decided to retain it both in the paper and in the software. Within the paper, it provides a useful reference geometry, and its availability in the software enhances the overall versatility of the detector array generator.

## 6. Results

The geometry constructed using the proposed method was subjected to a series of tests to evaluate its correctness. Based on the method’s output data, a three-dimensional model of the detector array was created. This model included supports for detectors positioned at appropriate angles, designed with the intention of being manufactured using 3D printing. However, fabricating a physical detector array proved too time- and resource-consuming to serve as a means of verifying subsequent stages of the method’s development and implementation. Therefore, a simulation program was created for this purpose.

Four flat detector arrays were prepared for both simulations and experimental validation on real hardware.

1. FG—flat geometry; created with detectors placed along the line.
2. AG—arc geometry; created using the arc-based method.
3. CLG—compact geometry, aligned to the left; created using the proposed method, with detectors aligned to the side closer to the radiation source.
4. CRG—compact geometry, aligned to the right; created using the proposed method, with detectors aligned to the side farther to the radiation source.

**Figure 6:**
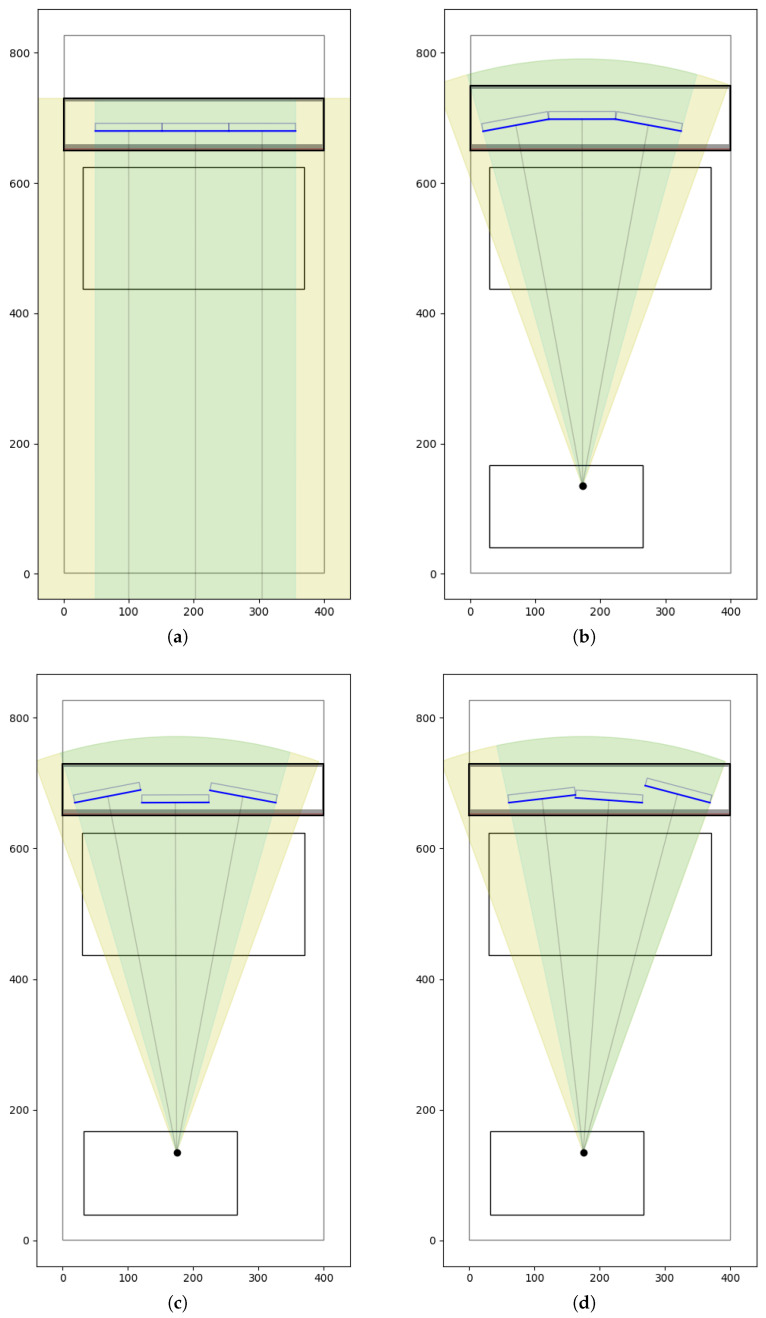
The detector array geometries that were used in the tests: (**a**) FG. (**b**) AG. (**c**) CLG. (**d**) CRG. The model shown in figure (**a**) was achieved by placing the radiation source at a distance many orders of magnitude larger compared to other parameters. This effectively created a linear array model without the need to introduce a new algorithm. The graphical convention in the diagrams matches that shown in Figure 3.

The schematic models of these geometries are shown in Figure 6. A scanner corresponding to the model shown in Figure 3 was also simulated. However, the physical dimensions of the test scanner, combined with the angular range limitations of the available X-ray source, prevented its experimental verification in the laboratory setting.

### 6.1. Simulation

The output of the simulation is a projection image equivalent to that which would be generated by a scanner configured with the specified geometry. The input data consists of the focal spot position *F* of the X-ray source, as well as the computed positions of the active regions for each detector, represented by the points $P_{L}$ and $P_{R}$ of the individual detectors.

In an actual scanner, each detector board records the projection signal accumulated over a unit of time as an *m*-element pixel vector. Analogously, in the simulation, the region between $P_{L}$ and $P_{R}$ of each detector is discretized into *m* sampling points: $p_{0},\ldots ,p_{m-1}$. The position of each point is determined by linear interpolation:

(4) $$p_{j}=\frac{m-j-\frac{1}{2}}{m}P_{L}+\frac{j+\frac{1}{2}}{m}P_{R},\;j=0,\ldots ,m-1,$$

where each point $p_{j}$ corresponds to a single pixel in the output image row. The parameter *m* represents resolution of the detector.

To determine the value of each detector pixel $p_{j}$, a segment is considered between the sampling point $p_{j}$, representing the center of the detector pixel, and the focal spot *F* of the X-ray source. The projection value is computed analytically based on the geometric intersection of the ray with the scanned object.

For each ray connecting *F* and each pixel $p_{j}$, the length of the segment lying within the object model is calculated. In this simulation, the object is modeled as an analytically defined geometry—specifically a sphere parameterized to fit the size of the scanner’s tunnel. The path length calculation is accomplished by solving the ray–object intersection problem and determining the corresponding entry and exit points of the ray within the sphere. The difference between the coordinates of these points yields the exact path length of the ray traversing the object. This resulting length directly defines the projection value assigned to pixel $p_{j}$ in the output image. The independence of each $p_{j}$ enables straightforward parallelization of the simulation process.

The simulation also checks whether the detector is partially obscured by an adjacent detector. In such a case, the pixels corresponding to the obscured area are assigned the projection value that represents complete attenuation.

To simulate the scanning of three-dimensional objects, a mechanism was implemented to emulate the movement of the scanning plane, analogous to the translation of an object on a conveyor belt in an actual scanner. While the *x* and *z* coordinates remain unchanged during the process, the *y* coordinate is varied within a defined range from $y_{\min}$ to $y_{\max}$. The data vector recorded for each consecutive $y_{i}$ position forms a new row in the resulting image.

The range and sampling density along the *y* axis must be chosen empirically to ensure that the final image preserves proportions consistent with those along the *x* axis. Similarly, in a physical scanner, the conveyor speed and detector sampling rate are adjusted to maintain the desired aspect ratio in the acquired image.

**Figure 7:**
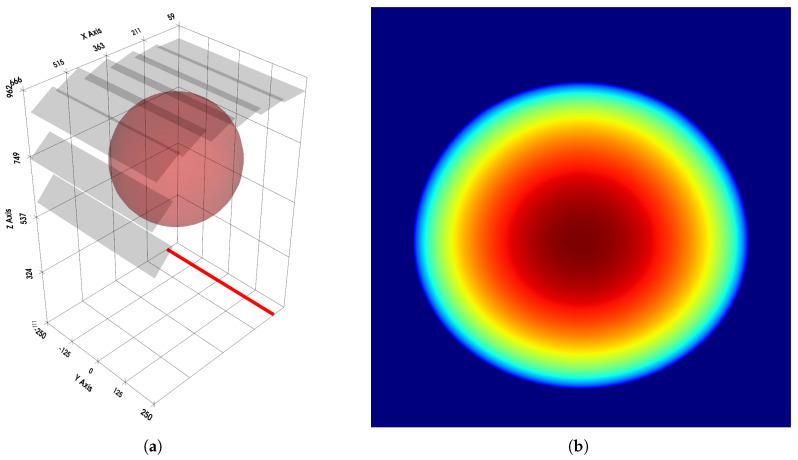
Simulation of a scan of a homogeneous solid sphere using the scanner geometry shown in Figure 3: (**a**) Three-dimensional model. The red line indicates the position of the radiation source, while the semi-transparent gray rectangles represent the detectors. Their extension along the *y* axis corresponds to the movement of the scanned object in this direction. (**b**) Projection on the detectors, using the jet colormap. The blue color corresponds to no attenuation, and the red color indicates maximal (relative) attenuation.

**Figure 8:**
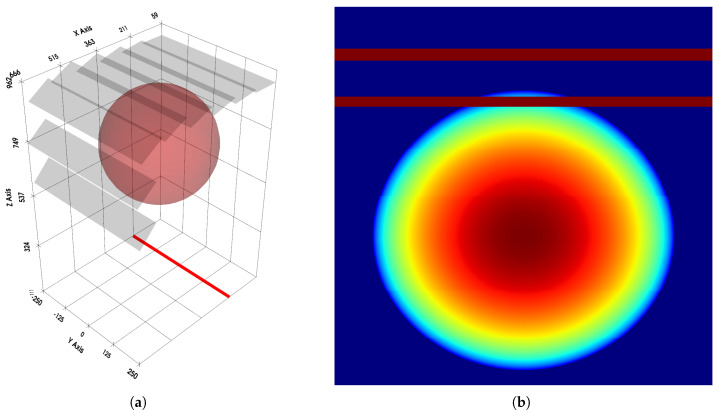
Simulation performed with a setup similar to that shown in Figure 7, but with the X-ray tube incorrectly shifted by 10 cm along the *x* axis, thereby violating the intended geometry: (**a**) Three-dimensional model. (**b**) Projection on the detectors. Discontinuities are visible between regions corresponding to individual detectors. Horizontal lines represent areas obscured by adjacent detectors.

All conducted tests demonstrated full continuity between detectors, regardless of the chosen geometry. Figure 7 presents a visualization of the simulated setup together with the obtained projection. The simulation can also be used to observe the impact of geometry design errors on the scanned object. A representative example of such an error is shown in Figure 8. The figure demonstrates that geometric inaccuracies affect detectors differently depending on their placement. This effect is further amplified by the shadowing of more distant detectors by closer ones, which was not accounted for in the simulation.

### 6.2. Experiments

A dedicated scanner was constructed to perform the evaluation. An XRB011 X-ray tube manufactured by Spellman, 475 Wireless Blvd, Hauppauge, NY 11788, USA was used. The tube emits a cone-shaped beam, which was collimated into a fan beam. The tube operates at a voltage of 80 keV, which is insufficient for use in baggage scanners due to limited penetration through metals. However, this does not affect the geometric correctness of the method.

**Figure 9:**
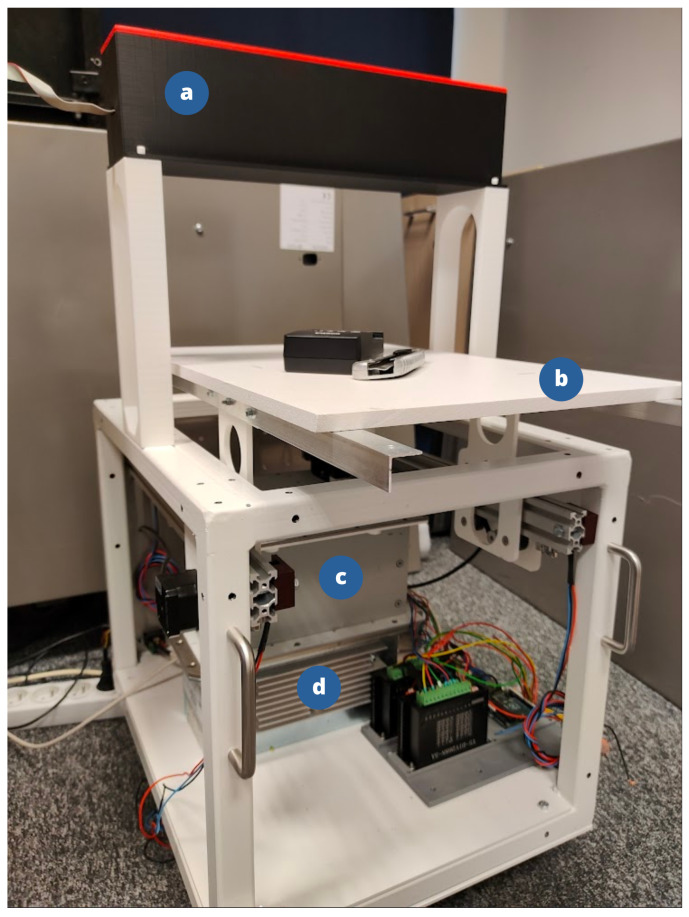
The experimental setup: (**a**) 3D-printed detector array. (**b**) Mobile platform. (**c**) Collimator. (**d**) X-ray tube.

**Figure 10:**
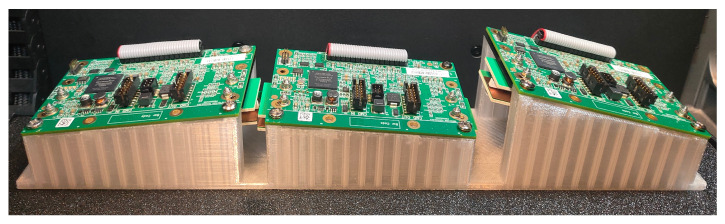
The 3D-printed array of the CRG geometry (as shown in Figure 6d) with mounted detector cards.

As detectors, D010812812B modules from Detection Technology Plc, A Grid, Otakaari 5A, 02150 Espoo, Finland were used. Each module contains two parallel detectors: the low-energy detector is positioned closer to the radiation source, while the high-energy detector is placed behind it and preceded by a copper filter that blocks a significant portion of the low-energy radiation. For the purposes of geometric calculations and image generation, only the low-energy detector was used. The image captured by the high-energy detector was not utilized in this paper. Each detector acquires a row of 128 16-bit pixels for each energy within a single integration interval. The experimental setup is depicted on the Figure 9 and the 3D-printed array is depicted on the Figure 10.

The effective length of a single detector module is 102.5 mm. The total length of the constructed detector array was limited by the working area of the 3D printer. The array was designed to be printed as a single piece, ensuring that any misalignments that could result from assembling multiple elements would not affect the outcome. As a result, the detector bar accommodated three detector cards.

The scanned object was placed on a platform moved by stepper motors, serving as a substitute for a conveyor belt. The resulting scanner was capable of recording images up to 40 cm in length. The scan width varied slightly depending on the detector array used, and was approximately 18 cm.

**Figure 11:**
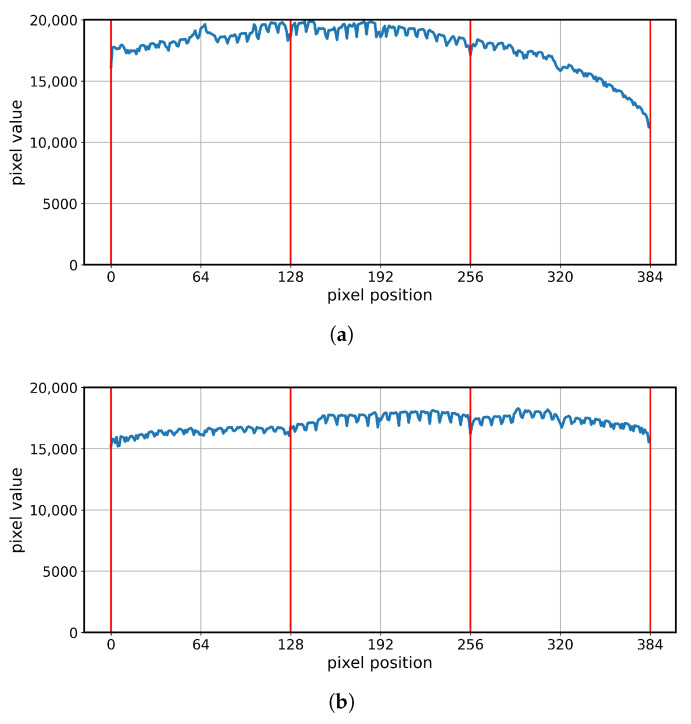

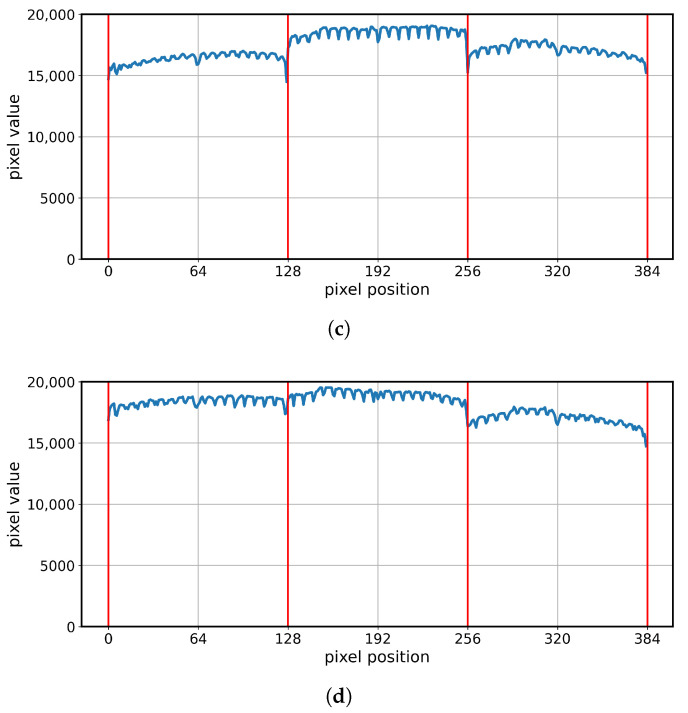
Detector unattenuated readout plots for the tested geometries: (**a**) FG, (**b**) AG, (**c**) CLG, (**d**) CRG. Red lines indicate the boundaries of the detectors.

For each prepared geometry, detector readout measurements were performed. The results were consistent with expectations and are illustrated in Figure 11. The FG and AG geometries demonstrated continuity of readouts between adjacent detectors, whereas the CLG and CRG geometries revealed step-like differences between them. At the same time, the AG, CLG, and CRG geometries exhibited stable readout levels along the entire length of each individual detector, while the FG geometry displayed a gradual monotonic change in this value.

Tests on physical detectors revealed an issue that did not occur in the simulation. Under certain configurations, a discontinuity appeared at the boundary between detectors, which was consistently present in all images for a given geometry.

**Figure 12:**
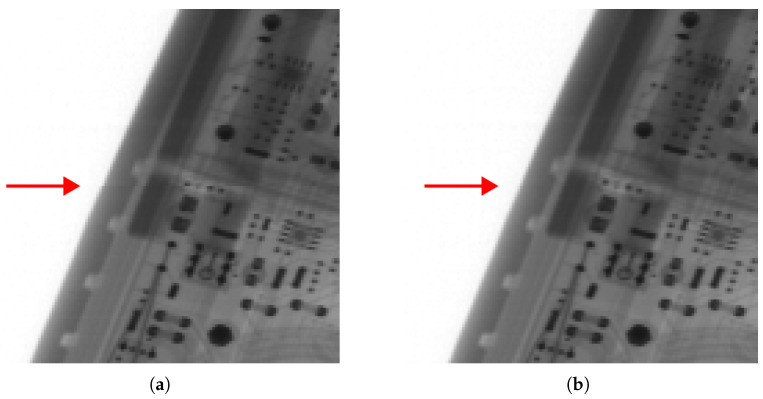
A 64 × 64-pixel area at the boundary between detectors on the image from the linear array. The boundary is marked with an arrow. In image (**a**), a discontinuity between the detectors is visible. The corrected version, obtained through translation and interpolation, is shown in image (**b**).

To investigate this phenomenon, a FG array was used, as it eliminates the possibility of geometry-related errors influencing the results. The discontinuity on the FG array is shown in Figure 12a. This issue was not observed at the other detector boundary within the same array. Swapping the detectors resulted in a continuous image, indicating that the problem was not related to geometry but rather to the physical characteristics of the detectors. Out of the seven detectors available for testing, five exhibited this issue.

The design of both linear and arc-shaped arrays does not allow for a physical solution by moving one or both detectors closer together, as they are already mounted with no clearance between them, leaving no room for adjustment. A potential solution for an array implementing the compact geometry would be to shift the detectors slightly. However, such shifting alters the angle of the moved detector, thereby compromising the designed geometry. While this change is negligible for a short array, with a larger number of detectors the error would accumulate.

The adopted method for achieving continuity involved separating the pixel blocks from adjacent detectors and filling the gap by linear interpolation of the separated edges. The result of this approach is shown in Figure 12b. In all tested geometry designs, the required translation did not exceed one pixel.

**Figure 13:**
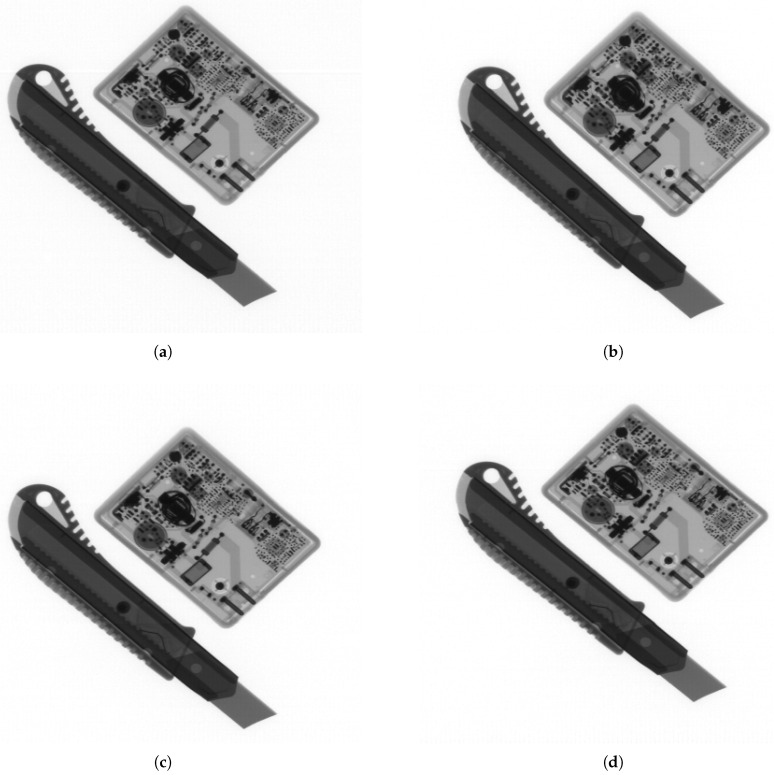
X-ray scans of a battery charger and a utility knife acquired using different detector geometries while keeping the scanned objects fixed. No discontinuities between detectors are visible in any of the images. Comparison between images reveals slight vertical translation and differences in object proportions, resulting from variations in geometry: (**a**) FG. (**b**) AG. (**c**) CLG. (**d**) CRG.

Tests conducted on all prepared geometries confirmed the correctness of each presented solution. The results are shown in Figure 13. No visible boundaries between detectors can be observed. The images differ slightly from one another, and none stands out as clearly superior. The geometries designed using the compact method enable flexible scaling of the detector array size, preserve minimal dimensions of a scanner, and ensure the correctness of the resulting images.

### 6.3. Runtime Performance

The proposed algorithm successfully reduced the complex task of detector fitting to a single-dimensional optimization problem involving the perpendicularity between the detector and the ray originating from the X-ray source’s focal spot. Both the translation and rotation variants utilize the bisection method to find the optimal position, yielding a time complexity of O(logn).

The bisection calculation is performed twice for each of the corner detectors and once for all other detectors in the array. The required number of iterative steps for each detector is determined by the tolerance parameter ε, which defines the precision of the method. Given that the detector array was modeled with the intention of being 3D printed, an accuracy of $\varepsilon =10^{-2}\;\mathrm{mm}$ was chosen. This sets the model’s required precision one magnitude higher than the resolution of the used printer (i.e., 0.01mm compared to 0.2mm). For a 100mm detector, this tolerance typically results in 9 to 10 iterations before the accuracy requirement is satisfied.

Performance tests were conducted on a machine equipped with an AMD Ryzen 7 5800X3D, 3.4GHz processor. For the benchmark model, which contained 410 detector cards, 100 measurements of the model generation time were carried out. The performance results are as follows:

- Model generation—the average time was 8.38ms, with a standard deviation of 0.19ms.
- Visualization—the average time was 337.95ms, with a standard deviation of 8.16ms.

Due to the integration of the algorithm with a user interface, the time required to generate the model’s visualization was also measured. When the option to redraw the visualization after every parameter change is enabled, the resulting latency is noticeable but does not impede the usability of the application.

The simulation algorithm’s time complexity is linearly dependent on the size of the resulting image. For an image of size 2048×2048 pixels the process took approximately 3 s.

## 7. Application

The software tool developed for this study is made available in a public repository [30]. This repository also contains the user instructions and system requirements. All examples detailed in this paper are bundled with the application. The software is written in Python 3.13 and uses Matplotlib 3.10 [31] for schematic drawings and PySide 6.10 for the graphical user interface. The simulation application is likewise written in Python, using PyVista 0.46 [32] for visualization.

## 8. Discussion

Most X-ray imaging studies either assume geometrically correct layouts or focus on post-acquisition corrections rather than pre-assembly geometry synthesis. In computed tomography (CT), geometric calibration is commonly achieved using many overlapping projections of the same object and specialized procedures to estimate source–detector misalignments and panel distortions [8,9,10,17,23]. These strategies rely on data redundancy that is unavailable in single-view line-scan baggage systems, where each image row corresponds to a distinct object slice and only one projection direction is present [16,17,18]. Methods that stitch or correct discontinuities across adjacent panels using image content (e.g., parallax-robust orthographic stitching [11]) can mitigate some artifacts, but they do not recover true geometry and are sensitive to scene content, occlusion, and object height variation.

The approach proposed in this paper formalizes detector placement as an explicit geometric optimization problem prior to assembly, thereby addressing the root cause of banding and tearing artifacts rather than compensating for them afterward. This study started based on the hypothesis that a continuous geometric model will remove inter-detector discontinuities. The simulation and laboratory results support this hypothesis. The comparative inclusion of flat and arc-based reference geometries aligns with established design choices in CT (arc-based) and line-scan radiography (flat), providing context for why compact arrays are suitable for baggage scanners, where available space is limited [15,19,23].

All tested geometries produced continuous images when implemented with sufficient precision, and the compact layouts generated by the proposed algorithm strike a favorable balance between geometric correctness and mechanical compactness (Figure 11, Figure 12 and Figure 13). The proposed compact geometry model is an implementation of a widely used design. However, the scientific literature lacked a detailed description of how such geometry is constructed. The presented algorithm is highly time-efficient, and the application allows predictable planning of the detector array relative to scanner dimensions.

### 8.1. Limitations

Although the simulation results provide a basis to assume that the algorithm will generate a correct array, the laboratory prototype’s scale (three modules) limits direct claims about long and non-linear arrays.

In dual-energy detectors, the two sensors are stacked vertically, and both have equal length. Due to this design, it is impossible to obtain a perfectly continuous image for both low- and high-energy channels simultaneously. Aligning the low-energy detectors leaves small gaps in the coverage of high-energy detectors. This solution was adopted in the proposed model. An alternative would be to align the high-energy detectors, but then low-energy detectors could collide, which might be resolved by partially overlapping adjacent detectors. This approach was not investigated in this study. Aligning images from both energy channels is a complex problem and is typically addressed using dual-energy registration algorithms [33].

### 8.2. Future Research

Experiments revealed that images exhibit pixel-level discontinuities at detector boundaries even in flat geometry. These gaps, shown in the figures, were manually corrected after acquisition. They are consistent across all images obtained with a given geometry and detector type, and they cannot be predicted during modeling. A valuable addition would be an automated procedure to correct these shifts based on a scanned phantom image.

From a usability perspective, the most obvious next step is to extend the application to generate a three-dimensional model ready for 3D printing or CNC machining instructions. Three-dimensional printing would enable easy prototyping of more complex constructions than those presented in this paper. However, professional deployment of such a solution would require addressing issues related to scaling, structural stability, and heat dissipation from detectors.

The author would also like to explore dual-row and stereographic configurations [27]. It is possible that AI-based interpolation methods [34] could be applied for partial 3D reconstruction. Currently, the method assumes that the detectors’ *y* axis is perpendicular to the xy plane. Introducing a tilt of all detectors relative to this plane could enable the creation of an array suitable for stereographic imaging.

## 9. Conclusions

This paper presents a parameterizable computational model for designing detector arrays in X-ray baggage scanners. The proposed method enables fast and precise calculation of detector positions and orientations, ensuring geometrically accurate image acquisition while minimizing scanner dimensions. Comparative tests using both simulation and physical prototypes confirmed that the generated geometries produce continuous, artifact-free images. The developed software tool significantly simplifies the scanner construction process, which is traditionally complex and error-prone when performed manually. This is achieved by automating the generation of accurate detector geometries and providing flexibility to adapt to different scanner sizes and configurations. Future work will focus on extending the tool to generate complete 3D-printable models and on incorporating detector arrangements that support stereographic imaging. Scanners constructed using the presented method will also serve as a platform for further research on X-ray image analysis and advanced material classification techniques.

## Data Availability

The original data used for the examples presented in this paper can be accessed in a public repository [30].

## Abbreviations

The following abbreviations are used in this manuscript:

CT	Computed tomography
FG	Flat geometry
AG	Arc geometry
CLG	Compact geometry, aligned to the left
CRG	Compact geometry, aligned to the right
